# Multivariate analysis applied to X-ray fluorescence to assess soil contamination pathways: case studies of mass magnetic susceptibility in soils near abandoned coal and W/Sn mines

**DOI:** 10.1007/s10653-024-01988-3

**Published:** 2024-05-02

**Authors:** Jelena Milinovic, Patrícia Santos, Helena Sant’Ovaia, Aurora Futuro, Carlos M. Pereira, Bramley J. Murton, Deolinda Flores, Manuel Azenha

**Affiliations:** 1 Chemistry and Biochemistry Department, Faculty of Sciences, CIQ‑UP, Institute of Molecular Sciences (IMS), University of Porto, Rua do Campo Alegre s/n, 4169‑007 Porto, Portugal; 2 Institute of Earth Sciences, Pole of University of Porto, 4169-007 Porto, Portugal; 3 Department of Geosciences, Environment and Spatial Planning FCUP, University of Porto, 4169-007 Porto, Portugal; 4 CERENA, Faculdade de Engenharia da Universidade do Porto, Rua Dr Roberto Frias s/n, 4200-465 Porto, Portugal; 5 NOC, National Oceanography Centre, European Way, Southampton, SO14 3ZH UK

**Keywords:** Metal contamination, Magnetic minerals, Geochemistry, XRF spectra, PCA and cluster analysis, Regression analyses

## Abstract

Determining the origin and pathways of contaminants in the natural environment is key to informing any mitigation process. The mass magnetic susceptibility of soils allows a rapid method to measure the concentration of magnetic minerals, derived from anthropogenic activities such as mining or industrial processes, i.e., smelting metals (technogenic origin), or from the local bedrock (of geogenic origin). This is especially effective when combined with rapid geochemical analyses of soils. The use of multivariate analysis (MVA) elucidates complex multiple-component relationships between soil geochemistry and magnetic susceptibility. In the case of soil mining sites, X-ray fluorescence (XRF) spectroscopic data of soils contaminated by mine waste shows statistically significant relationships between magnetic susceptibility and some base metal species (e.g., Fe, Pb, Zn, etc.). Here, we show how qualitative and quantitative MVA methodologies can be used to assess soil contamination pathways using mass magnetic susceptibility and XRF spectra of soils near abandoned coal and W/Sn mines (NW Portugal). Principal component analysis (PCA) showed how the first two primary components (PC-1 + PC-2) explained 94% of the sample variability, grouped them according to their geochemistry and magnetic susceptibility in to geogenic and technogenic groups. Regression analyses showed a strong positive correlation (*R*^2^ > 0.95) between soil geochemistry and magnetic properties at the local scale. These parameters provided an insight into the multi-element variables that control magnetic susceptibility and indicated the possibility of efficient assessment of potentially contaminated sites through mass-specific soil magnetism.

## Introduction

Soil magnetic susceptibility is a physico-chemical property that describes the magnetic response of soil to a uniform external magnetic field (Evans & Heller, 2003). The volume magnetic susceptibility (*κ*) is the ratio of the magnetization (*M*) to the magnetic field (*H*). Since both parameters, *M* and *H,* have the same units (A m^−1^), their ratio, *i.e.*, the value obtained for the* κ* is dimensionless and difficult to compare with the values reported in the literature. Therefore, it is more convenient to use the mass or specific magnetic susceptibility (*χ*), which represents the ratio between the *κ* and density (*ρ*) of soil samples. Results of *χ*, which refer to the mass of soil samples (expressed in units of reciprocal density, m^3^ kg^−1^), are more readily available in the literature and more useful for comparison (Mullins, 2006). Conventional measurements of soil mass magnetic susceptibility (*χ*) usually performed under a weak magnetic field, give more reliable results because at lower levels of *H* (< 1000 A m^−1^), the magnetization is directly proportional to the applied magnetic field (Evans & Heller, 2003).

The magnetic properties of soil are primarily due to the presence of ferromagnetic minerals (maghemite, magnetite, and hematite) and some other minerals with paramagnetic behavior (e.g., biotite and ilmenite) (Dekkers, 1997; Magiera et al., 2006; Mello et al., 2020). Other minerals, such as silicates, have less influence on the soil magnetic susceptibility, while their presence and composition can indicate the lithology of the soil’s parent rock. In addition to the bedrock contribution, anthropogenic contamination can also affect soil magnetic susceptibility through pathways such as atmospheric deposition and precipitation, road and rail traffic, fires, disposal of waste materials, cement and metallurgical industries, as well as particulates that can be very magnetic and which could indicate a potential risk for human health (Dellibecque et al., 2022; Lu et al., 2012). Soil magnetic susceptibility is, therefore, a sensitive parameter that depends on numerous factors whcih, if they can be separated and identified, can be used as an indicator of environmental contamination (Pan et al., 2019; Wang et al., 2018).

In the case of mining sites and their surrounding environments, the increase in magnetic susceptibility is positively correlated with the presence of high contents of certain metal species. For example, Attoucheik et al. (2017) found a positive correlation between magnetic susceptibility and Pb (at concentrations lower than 500 mg kg^−1^) in the topsoils near a Pb–Zn mine (in Algeria); Nahan et al. (2023) observed that magnetic susceptibility was correlated with Fe and Sc concentrations in gold mining sites (Indonesia); Ma et al. (2023) concluded that Pb and Co pollution, found in dust from the extremely arid city of Dunhuang (NW China) and mainly derived from coal mining and coal combustion, coincided with mass specific magnetic susceptibility of135 × 10^−8^ m^3^ kg^−1^.

The underlying processes behind the correlations between magnetic susceptibility and the presence of these contaminants are not straightforward. Recent studies showed that correlations between magnetic susceptibility and metal contaminants were largely related to geogenic or anthropogenic (technogenic) compositional specifics of the respective mining site (Bourliva et al., 2017; Chakraborty et al., 2023; Magiera et al., 2023; Shaheen & Iqbal, 2018; Szuszkiewicz et al., 2021). For example, influx of paramagnetic mineral particles containing the correlated elements, or the adsorption of correlated elements onto paramagnetic dust particles.

In this research, we report how multivariate analysis (MVA) identifies complex univariate data correlations between the mass magnetic susceptibility and the concentration of individual elements, for soils from one or more mining sites of similar type. Our study uses X-ray fluorescence (XRF) spectroscopy data acquired by portable XRF instruments, that are widely used among geologists and environmental scientists to characterize a wide range of elemental concentrations (Agyeman et al., 2021; Bosco, 2013; Jang, 2010). Given the previous studies, it is reasonable to expect that XRF spectroscopic data encrypt information directly related to magnetic susceptibility, which could be deconvolved using regression MVA. Detailed explanations of the advantages of applying MVA to XRF spectroscopic data in soil survey (e.g., for rapid soil characterization), are given elsewhere (dos Santos et al., 2020; Milinovic et al., 2023; Morona et al., 2017; Panchuk et al., 2018; Rocha et al., 2019). If successful, MVA applied to both XRF and magnetic susceptibility data from soils could provide insights into the pathways that enable contaminants to enter the environment. Furthermore, the MVA approach would allow field scientists to estimate potentially contaminated sites via mass magnetic susceptibility (*χ*) alone, enabling rapid scanning for the contamination.

The main objectives of this work were the following: (i) to obtain qualitative geochemical signatures encrypted in soils using XRF spectroscopic data that can be correlated with magnetic properties of the soil; (ii) grouping of different soil types according to geochemical signatures by classification MVA; (iii) application of regression MVA to obtain XRF–MVA models, the interpretation of which can clarify the soil contamination pathways. These objectives were achieved by testing soils from three abandoned mining areas (in NW Portugal): two coal mines (São Pedro da Cova and Fojo) and a W/Sn mine (Regoufe).

## Materials and methods

### Geological setting of study areas and soil sampling

**Fig. 1:**
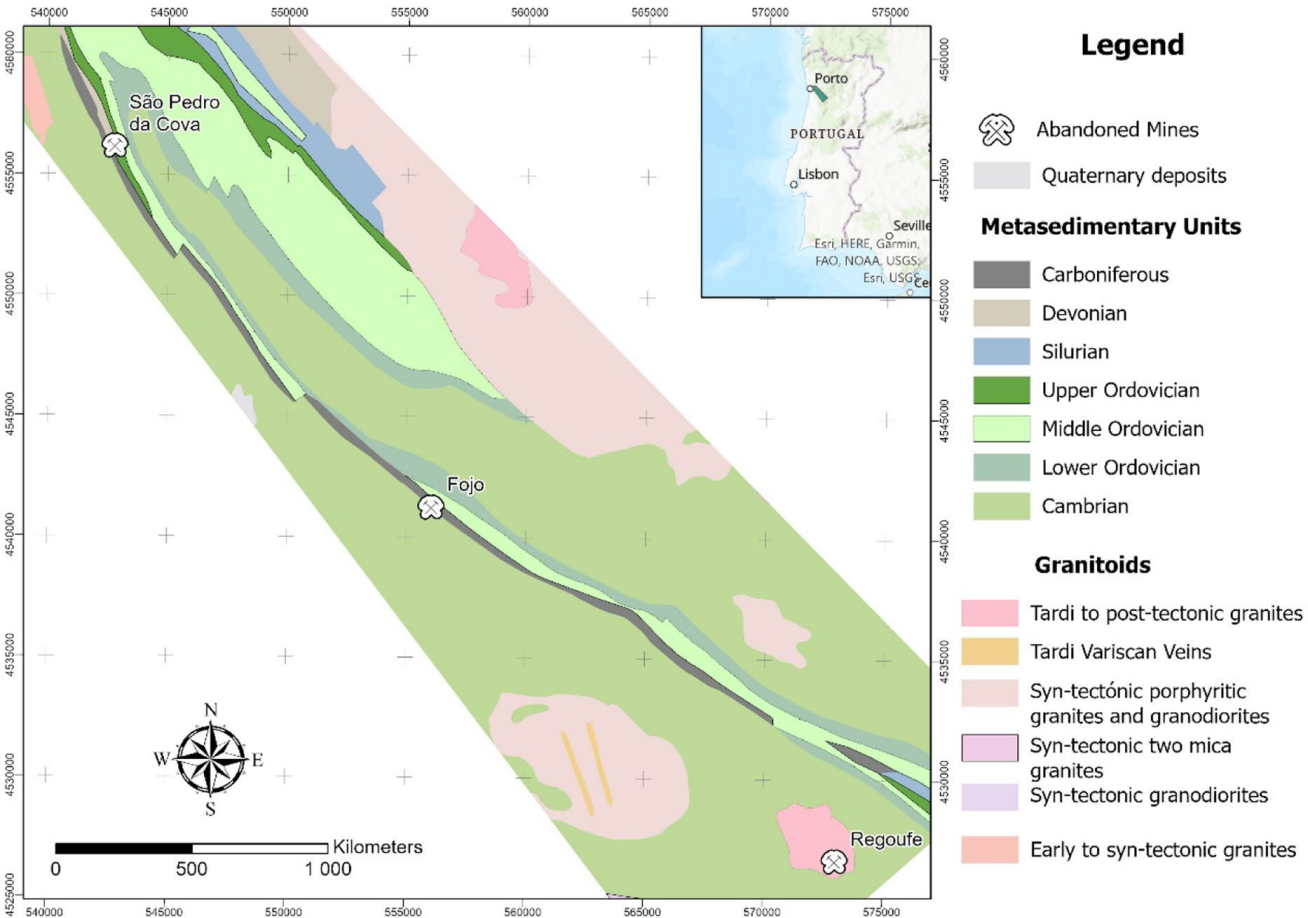
Geologic setting of the studied pilot areas: São Pedro da Cova, Fojo, and Regoufe

Soil samples used in this study were taken from three different study areas in NW Portugal (Fig. 1). These sites comprise of three abandoned mines: São Pedro da Cova (41° 09′ 25″ N; 08° 30′ 06″ W), a coal mine located in Gondomar; the Fojo mine (41° 09′ 54″ N; 08° 20′ 28″ W), which is part of the Pejão coal mining complex, located in Castelo de Paiva, and the Regoufe (40° 52′ 44″ N; 08° 08′ 06″ W), a wolframite/cassiterite mine located in Arouca.

Both São Pedro da Cova and Fojo mines exploited anthracite A (ISO 11760:^2005^) from the Douro Carboniferous Basin which was later used in energy production. This sedimentary basin, dating from the Upper Pennsylvanian (Correia et al., 2018; Lemos de Sousa & Wagner, 1983) was the most important coal reserve in Portugal, extending NW–SE, from São Pedro de Fins to Janarde, over a distance of approximately 53 km and between 30 and 250 m thick (Pinto de Jesus, 2019). The São Pedro da Cova mine was closed in 1972, after working for almost two centuries. The Fojo mine was exploited until 1994, after which the spoil-heaps burned between 2007 and 2019, after being ignited by wild fires in the surrounding vegetation. The combustion of the spoil-heaps was only extinguished after the intervention of a state company that applied a cooling agent combined with the remobilization of the materials affected (Santos et al., 2023).

Regoufe is a wolframite/cassiterite (W/Sn) mine, that started operating in 1915 (motivated by World War 1). During World War 2 it saw a significant increase in production but was closed in the 1970s. The mine is located along the SE border of the Regoufe pluton, a 6 km^2^, NW–SE elongated circular muscovite-albite porphyritic granite batholith, with a whole-rock Rb–Sr age of 280 ± 8 Ma (Fig. 1). Several tungsten-rich deposits occur in this granite body, where they are host in 10–20 cm thick cross-cutting quartz veins (Pereira et al., 2007; Van Gaans et al., 1995)and contain wolframite, cassiterite, arsenopyrite, and minor amounts of pyrite, sphalerite, apatite, and beryl (Sant’Ovaia et al., 2023).

In São Pedro da Cova and Fojo, the soil samples collected are essentially the result of the pedogenesis of underlying metasedimentary rocks of Cambrian to Carboniferous ages, while in Regoufe, the soil is derived from the granitic substrate (Fig. 1).

The mines at the study sites have undergone little intervention or restoration since their abandonment, apart from the extinguishing of the burning spoil-heaps at Fojo. Therefore, the composition and geochemical signature of surrounding soil reflects a complex origin with input from the local bedrock and input from anthropogenic effects including mining activities.

**Fig. 2:**
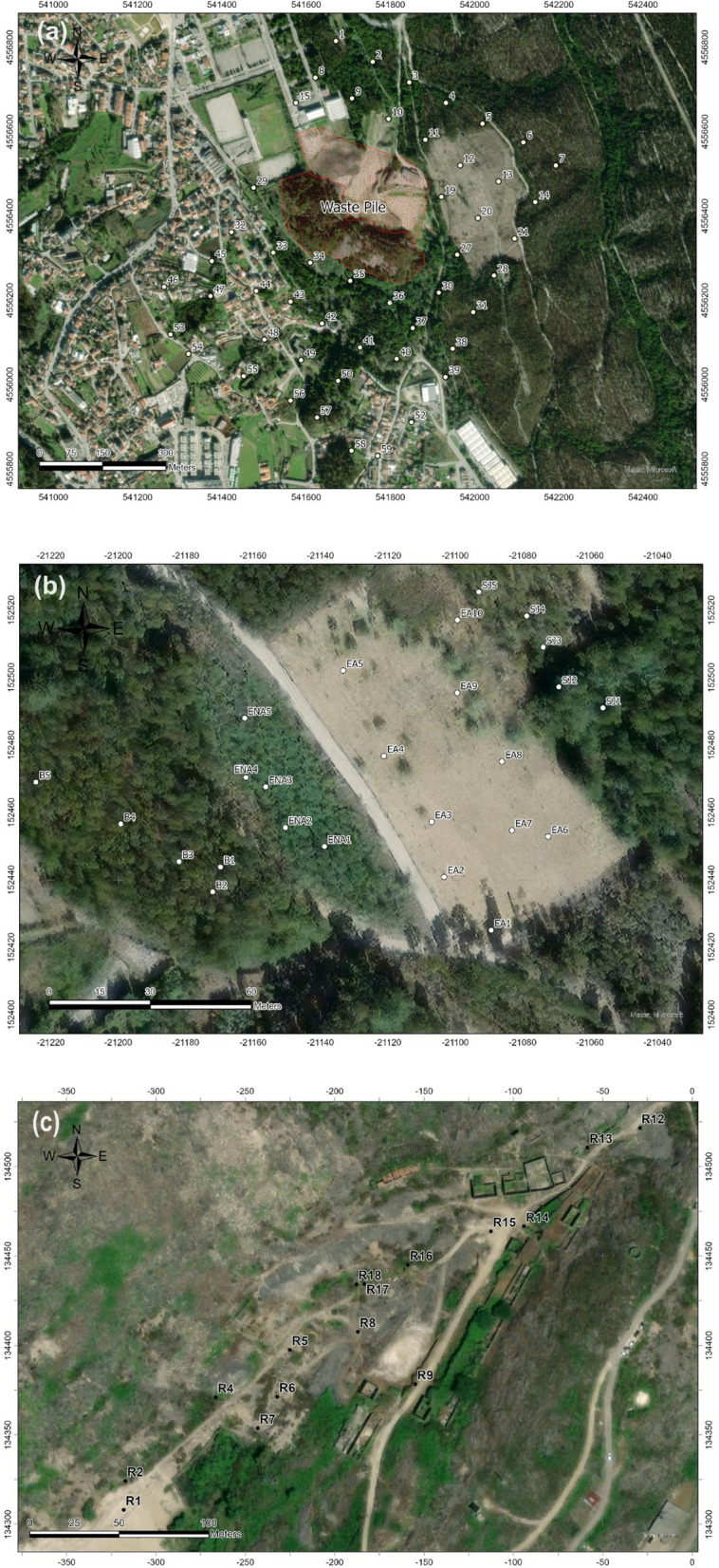
Maps of the sample locations in studied pilot areas: São Pedro da Cova (**a**), Fojo (**b**), and Regoufe (**c**)

A total of 90 heterogeneous topsoil samples were collected from three mining areas and chosen as a purposeful database that will serve better to test the applicability of MVA (at local vs regional level of tested mining areas). Each sample was collected from a single point at a depth of less than 20 cm (topsoil) from the three abandoned mining areas: São Pedro da Cova (n = 50), Fojo (n = 25), and Regoufe (n = 15) (Fig. 2).

It is not possible to detect all 50 points in this picture above!

There are 24 points and not 25 like expected!

I recommend that you put letters (a), (b), (c) in these pictures.

The soil samples surrounding the waste pile in São Pedro de Cova were collected systematically at approximately 100 m distance (Fig. 2a). The sampling sites covered a complex area including forest, urban, and industrial zones, as well as small landfills.

The soil samples from the Fojo mining area were taken from different forest areas in and around the abandoned coal mine waste pile (Fig. 2b). Some of the soil samples were collected from the areas affected by remobilized burnt spoil-heaps (EA, 1–10), as well as from outside the affected areas (ENA, 1–5). A number of samples were also collected from an area downslope from the spoil areas (SJ, 1–5), and which were affected by the runoff from the waste pile. Reference samples were taken uphill of the waste pile, in the forest area (B, 1–5), thus representing a geochemical baseline for the soils unaffected by the mining deposition. The samples in the Regoufe mining area were taken from a valley next to the old mining facilities (Fig. 2c). Extensive descriptions of the soil sampling, pre-treatments, and homogenization of representative soil samples are provided elsewhere (Sant’Ovaia et al., 2023; Santos et al., 2023).

### Analysis of soil samples

The soil samples from three mining areas were characterized as previously explained (Ribeiro et al., 2011; Sant’Ovaia et al., 2023; Santos et al., 2023). The mass magnetic susceptibility was determined in studied soil samples by their exposure to a magnetic field of 300 A m^−1^ on a KLY-4S Kappabridge instrument (Advanced Geoscience Instruments Co., Czech Republic). The KLY-4S Kappabridge is a high-sensitive laboratory instrument, operating at a frequency of 875 Hz and power consumption of 45 VA. Three measurements were performed for each sample and the mean values are presented in the results.

The same soil samples were analysed by energy dispersive X-ray fluorescence spectrometer (X-MET 7500, the handheld XRF Mining Analyzer, Oxford Instruments, UK) equipped with a rhodium anode X-ray tube working at 40 kV voltage and 10 µA current (Figueiredo et al., 2019). Spectroscopic data at broad energy level (1–35 keV) were obtained in Mining Mode, and averaged values across the three entire-spectra were extracted for each sample and exported for further treatment by MVA.

Due to XRF spectral interferences (overlapping signals) and the high limit of detection for the elements analysed by XRF, the concentrations of soil elements of geochemical importance in this study (As, Fe, Pb, Rb, Sn, Sr, Ti, Y, Zn, Zr) were independently obtained by inductively coupled plasma mass spectrometry (ICP-MS). These values served as references in the explanation and discussion of the obtained XRF–MVA results. Official ICP-MS analyses were performed in Accredited Laboratory N° 270 in Canada (Bureau Veritas Labs, Vancouver). Samples were digested in HF-HClO_4_-HNO_3_ and subsequently the dissolved residue was analyzed by ICP-MS. Blanks, random duplicate samples and three certified reference materials (OREAS: 25A, 45E, and 45H), were used for the quality control of analytical procedure. The limits of detection (LOD) for the analyzed elements were in the following order: 0.000002% for Pb; 0.00002% for As, Zn, and Zr; 0.00001% for Rb, Sn, and Y; 0.0001% for Sr; 0.001% for Ti; and 0.01% for Fe. Full details of the sample preparation, operating conditions and quality control of the ICP-MS analyses are described in detail elsewhere (Sant’Ovaia et al., 2023; Santos et al., 2023).

### Multivariate analysis applied to X-ray fluorescence spectroscopic data

Quantitative data encrypted in XRF spectra (K-alpha and K-beta peak energies for each of the elements of interest) of studied topsoil samples (n = 90) were analyzed by multivariate analysis (MVA) using the software application Unscrambler. Unsupervised classification MVA, principal component analysis (PCA) and cluster analysis were applied to entire XRF spectroscopic data to reduce their dimensionality (by excluding sample outliers), and identifying correlated parameters to arrange the samples into empirical groups (Brereton, 1990). Classification and regression MVA were applied to the raw and transformed XRF spectra of the studied samples.

The XRF spectra were transformed using several techniques, such as normalization by the peak areas (that correlate with each element, background correction, Savitzky-Golay smoothing, and orthogonal signal correction (OSC). Applied transformation tests showed that only OSC, a mathematical treatment that removes factors unrelated to the sample variable concentration and orthogonal to the reference values of the variable of interest, was suitable for reducing side effects and larger variations in spectra (Blanco et al., 2001; Malmir et al., 2019; Zhang et al., 2013). For soil geochemistry-magnetism correlation, partial least square (PLS) regression was applied to XRF spectroscopic data versus the mass magnetic susceptibility (*χ*). Calibration was performed with 2/3 of samples while validation of the PLS regression was tested with the remaining 1/3 of the samples, a as the most effective method for dealing with heterogeneous samples of smaller size (Kardanpour et al., 2014; Martens & Naes, 1998; Martins et al., 2010; Nengsih et al., 2019). The relevant parameters used to evaluate the regression were the correlation coefficient (*R*^2^), which describes the linearity between the XRF-spectra and reference mass magnetic values in the plot, and the root mean square error (RMSE), which is a measure of dispersion for the samples about the regression line. These two parameters are optimal indicators for describing the goodness of fit in linear regression (Engelen et al., 2004). To identify the most important geochemical variables of the best fitted PLS regressions, the magnitudes of the weighted regression coefficients (B_w_) were examined (Ng et al., 2022). The regression coefficients (B_w_) were calculated incorporating one factor (or principal component) thus summarizing the relationship between the most important variables and the response.

For PCA, cluster analysis, OSC transformation, and PLS regression analyses, the nonlinear iterative partial least square (NIPALS) algorithm was chosen, as the most appropriate algorithm for the smaller sample size (Nengsih et al., 2019). Outliers were filtered according to Hotelling’s t^2^ statistic (*P* < 0.05).

## Results and discussion

### Variability of mass magnetic susceptibility in studied mine soil samples

The studied soil samples show a wide range of mass magnetic susceptibility (*χ*), *i.e.*, from 2.15 to 1003, from 13.7 to 749, and from 2.17 to 477 (× 10^−8^ m^3^ kg^−1^) in the São Pedro da Cova (Santos et al., 2023), Fojo, and Regoufe (Sant’Ovaia et al., 2023) mining areas, respectively (Table 1). These results show that samples in São Pedro da Cova (n = 50) had the highest variability in magnetic susceptibility, which differed by four orders of magnitude. For the samples surrounding the self-burning coal waste pile, the highest values were observed upstream of the waste pile pointing to possible origins for the ferrimagnetic minerals not related to the mine waste (Santos et al., 2023).

**Table 1:** Obtained values of the mass magnetic susceptibility (*χ*) in studied soils from São Pedro da Cova, Fojo, and Regoufe mining areas

Soil samples origin	n	Range of *χ* (× 10^−8^ m^3^ kg^−1^)
São Pedro da Cova	50	2.15–1003
Fojo (label)	25	13.7–749
Burned waste piles (EA)	10	285–749
Unburned waste piles (ENA)	5	20.4–98.4
Downhill area (SJ)	5	134–244
Forest (B)	5	13.7–65.8
Regoufe	15	2.17–477

The values of mass magnetic susceptibility in the Fojo samples from the burned mine waste area (EA, Fig. 2b), showed the highest values, ranging from 285 to 749 (× 10^−8^ m^3^ kg^−1^). The next highest values, in a range from 134 to 244 (× 10^−8^ m^3^ kg^−1^) are from samples (SJ) taken downhill and in the runoff affected area from the burned mine waste. Samples from unburned waste piles (ENA), have values that are an order of magnitude smaller than the burned mine waste samples, *i.e.*, from 20.4 to 98.4 (× 10^−8^ m^3^ kg^−1^) (Table 1). The reference soil samples from the forest uphill and away from the mined area (B) showed the lowest values, ranging from 13.7 to 65.8 (× 10^−8^ m^3^ kg^−1^), and overlapping with the unburned waste pile samples (ENA).

The high variability of magnetic susceptibility in the studied soil samples from the three abandoned mining areas (São Pedro da Cova, Fojo, and Regoufe) indicated that ferromagnetic minerals were present in different concentrations in the studied soil samples. Variability appears to relate to location with respect to sources of contamination, especially for the Fojo area, where there is better control on the location of the contaminated and uncontaminated sites (Flanders, 1994; Santos et al., 2023). To better explain the variability of the soil mass magnetic susceptibility, and hence the source of potential contamination, we made a MVA of the qualitative geochemical information encoded in the XRF spectra and compared them to the reference ICP-MS data.

### Soil geochemistry near abandoned coal and W/Sn mines

In our study, the reference ICP-MS results showed wide ranges of element concentrations in the studied soil samples (Table 2). Arsenic was found at similar concentrations in samples from both the coal mine regions (São Pedro da Cova and Fojo), while in the W/Sn (Regoufe) mine As contents reached a value of up to 1%. In continental crust, As is found primarily in an inorganic form (arsenic compounds) associated with igneous and sedimentary rocks (Biney et al., 2022), at average concentrations of 0.0002% and 0.0013%, respectively (Turekian & Wedepohl, 1961). Hence, the elevated As concentrations are likely to reflect contamination from mineralisation and mining. Besides the geogenic contribution, in non-mining areas, As can originate from several anthropogenic sources such as fertilizers applied to agricultural soils. This may explain the As levels in the São Pedro da Cova urban zone (Fig. 2). In these samples, As was found at levels up to 0.006% (Table 2).

**Table 2:** Obtained ICP-MS concentrations of elements in studied soils from São Pedro da Cova, Fojo, and Regoufe mining areas

Element	Range of element concentrations (%)		
	São Pedro da Cova	Fojo	Regoufe
	n = 50	n = 25	n = 15
As	0.001–0.006	0.001–0.008	0.075–1
Fe	1.08–9.39	1.17–7.09	0.85–5.99
Pb	0.002–0.016	0.002–0.012	0.001–0.198
Rb	0.007–0.029	0.004–0.017	0.043–0.082
Sn	0.0001–0.002	0.0002–0.001	0.005–0.010
Sr	0.003–0.069	0.005–0.014	0.002–0.005
Ti	0.077–0.520	0.154–0.375	0.042–0.066
Y	0.0005–0.002	0.001–0.002	0.0005–0.001
Zn	0.002–0.030	0.001–0.015	0.008–0.050
Zr	0.004–0.017	0.006–0.012	0.002–0.007

Iron has very high concentrations in the coal and W/Sn mine areas, reaching maximum concentrations of 9.39%, 7.09%, and 5.99% in samples from the São Pedro da Cova, Fojo, and Regoufe mines, respectively. Although the raw coal has relatively low concentrations ofFe, combustion (e.g., of the self-burning waste piles) can concentrate non-volatile elements, such as iron oxide (Santos et al., 2023). Other metallic elements (Pb, Rb, Sn, Sr, Y, Zn and Zr) were found in similar concentrations in the coal mine samples, reaching a maximum level of up to 0.07%. Similar concentrations were observed in the Regoufe W/Sn mine area (with the exception of Pb, which was found in concentrations up to 0.2%). The maximum Ti content in the soil was equal to 0.52% and 0.37%, in São Pedro a Cova and Fojo, respectively, while in the Regoufe soils its concentration was less than 0.07%.

### Qualitative geochemical information encrypted in soil X-ray fluorescence spectra

**Fig. 3:**
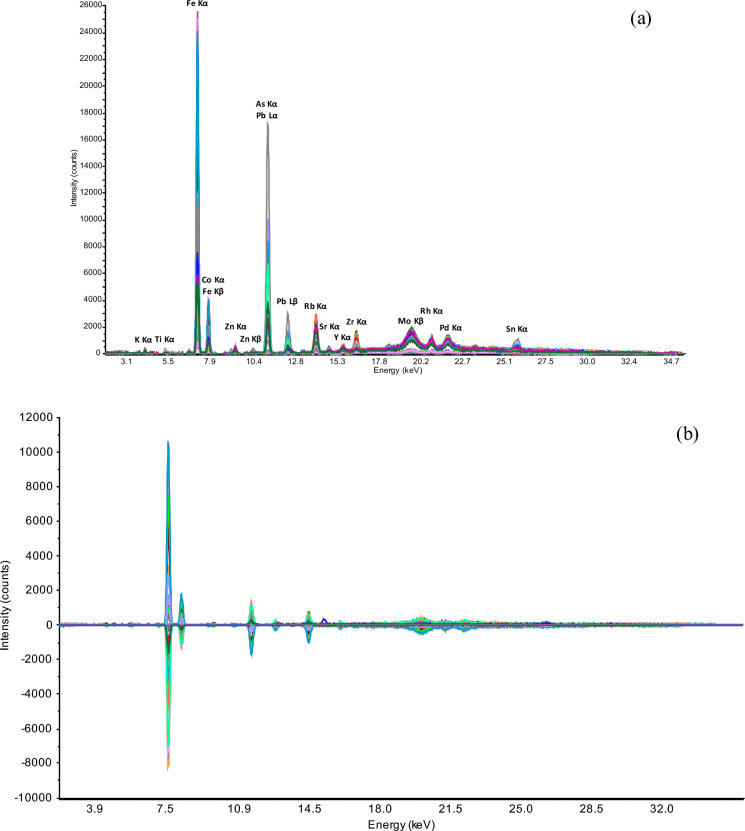
Mean-plot of spectra from studied soils (n = 90) around three mining areas: raw XRF (**a**) and transformed OSC-XRF spectra (**b**)

Iron and some other elements, typical in the studied mining areas (As, Pb, etc.) were detected by XRF spectral analysis, at characteristic energy levels (Fig. 3a).

The raw XRF-spectra showed the most intense peaks at 6.4 (K_α_) and 7.1 keV (K_β_), corresponding to Fe, while Rb, Sr, Zr, and Y at 13.4, 14.1, 14.9, and 15.7 keV, showed respective smaller peaks due to their lower concentrations in these samples (Table 2). The intense peaks of As and Pb at 10.5 (K_α_) and 11.7 keV (K_β_) were typical for the samples collected in the Regoufe (W/Sn) mining area, where these elements were found in high concentrations (e.g., As up to 1%). Broad peaks at 19.2, 20.2, 21.1, and 25.2 keV correspond respectively to Mo, Rh, Pd, and Sn.

After applying the OSC transformation (relative to the reference soil mass magnetic susceptibility values), the spectral differences have been minimized (Fig. 3b). The OSC pretreatment reduced the noise level and acted as a filter by eliminating all the information irrelevant to the target variable, *i.e.*, mass magnetic susceptibility (*χ*) and interfering features, improving the quality of spectroscopic data in the entire energy-range. As a result, peaks unrelated to mass magnetic susceptibility became much smaller (compared to their original intensities), but still present in the spectra (Blanco et al., 2001; Wu et al., 2006).

### Soil grouping based on geochemical signatures by principal component analysis

**Fig. 4:**
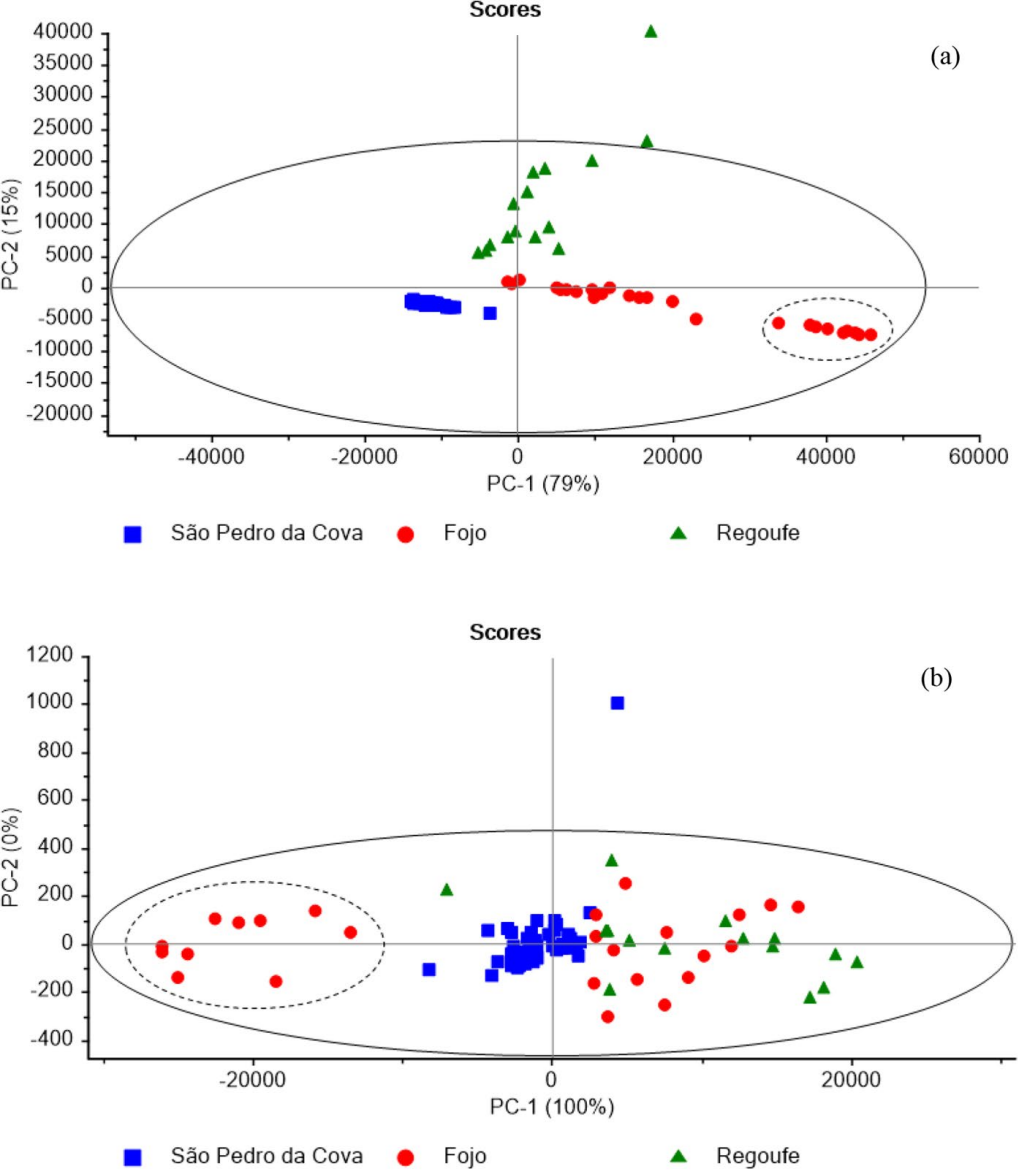
PCA applied to: raw XRF (**a**) and OSC-XRF (**b**) spectra of studied soils (n = 90) around three mining areas (São Pedro da Cova, Fojo, and Regoufe). The scores plot of the first two principal components shows that ten self-burning subset of samples of Fojo (bounded by a dashed line) were separated into an independent group

After applying PCA to raw XRF spectroscopic data of the studied soil samples (n = 90) three groups, each corresponding to a different mining area, have merged (Fig. 4a). Two-dimensional PCA also performed well, with PC-1 explaining quantitatively (79%) the variability of the spectroscopic data. The São Pedro da Cova and Fojo samples are distributed along the PC-1 axis, while the Regoufe samples are distributed along PC-2. The homologous distribution of samples from São Pedro da Cova and Fojo can be explained by the similarity in the geochemical signatures of these coal mining areas, *i.e.*, the soils of both pilots originated from similar metasedimentary units (Fig. 1) and were influenced by similar coal mining effects. In contrast, the Regoufe samples (W/Sn mine) had a distinctive signature because the soils derived from the Regoufe granite, and were later affected by tungsten mining activities, which form different rock type to the sedimentary coal sequences. The PCA also discriminates between a subset of samples from the Fojo mining area, designated as the self-burning waste pile (EA), and bounded by a dashed line in Fig. 4a, and other samples from the same mining area. The separate distribution of Fojo samples from the self-burning mine waste pile can be explained by the samples having a higher magnetic susceptibility (Ribeiro et al., 2014; Santos et al., 2023). Multivariable geochemical information, encrypted in the XRF spectra of the studied soil samples, can distinguish the different origins of the soil samples including those with distinctive and magnetic susceptibility.

The PCA applied to the raw XRF spectra (Fig. 4a) shows better discrimination of different mine samples, explaining 94% of the sample variability (PC-1 = 79% + PC-2 = 15%). The results of the PCA applied to OSC-XRF spectra (Fig. 4b) showed that the first component explained quantitatively the variability of samples (PC-1 = 100%) and all soil samples are essentially distributed along the PC-1 axis, less separated into discrete groups of mine origin, but rather in relation to the magnetic susceptibility values. The self-burning waste pile soil samples (bounded by a dashed line) from the Fojo were more anti-correlated with other samples from the same mining area groups (Fig. 4a, b). This also coincides with the higher magnetic susceptibility of the burnt waste-pile soils and hence indicates their unique origin. One sample from the São Pedro da Cova (19) was considered a significant outlier (visualized as the point in the top far away from the scores plot, Fig. 4b), due to its very high Fe content, equal to 9.39% (Table 2), and hence was excluded before PLS regression analysis was run.

### Cluster analysis of soils from São Pedro da Cova mining area

**Fig. 5:**
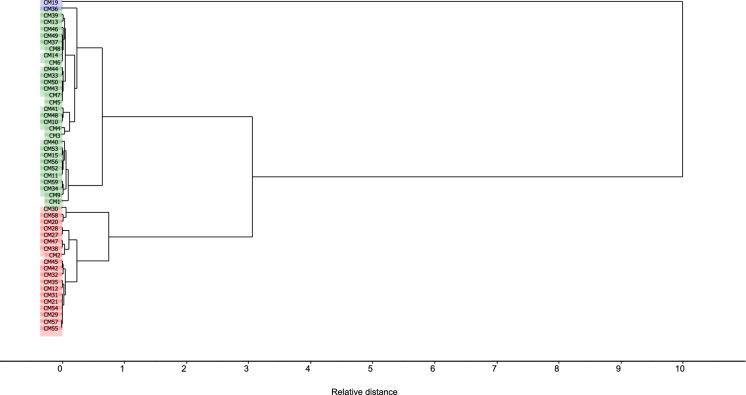
Cluster analysis using squared Euclidean distance, applied to samples (n = 50) of São Pedro da Cova complex area. After excluding one sample (19), as an outlier, a dendrogram illustrates the arrangement of two major clusters based on the relative distance of the corresponding XRF spectra: a cluster 1 in green (n = 30) and the cluster 2 in red (n = 19)

The interpretation of geochemical signatures in soil samples from São Pedro da Cova mine area (including forest, urban and industrial zones and small landfills), was facilitated by cluster analysis directly applied to raw XRF spectroscopic data of the entire set of soil samples. The procedure of cluster analysis is designed to discover natural clusters within a complex data set that would not otherwise be apparent. In this case, the dendrogram (produced using squared Euclidean distance) confirmed that one sample (19) was grouped as an independent cluster (i.e., an outlier, Fig. 5) as previously shown by PCA (Fig. 4b). Despite its maximum Fe content, an outlier sample showed a very low mass magnetic susceptibility (*χ* = 11 × 10^−8^ m^3^ kg^−1^), which can be explained by the presence of antiferromagnetic mineral, for. ex., goethite (Guyodo et al., 2003; Mello et al., 2020).

The two remaining statistically meaningful clusters, labelled as clusters 1 and 2 are shown in different colors (green and red, respectively) in Fig. 5. Cluster 1 (n = 30) was composed of samples from the forest and small landfill areas of the São Pedro da Cova mine sites (e.g., 3–11, 13–14, 39–41, 48–52, etc., Fig. 2a), while cluster 2 (n = 19) contained samples mostly around the waste pile, and from urban and industrial areas (e.g., samples 2, 21–22, 27–32, 54–55, etc., Fig. 2a). The cluster 1 (n = 30) was characterized with lower Fe (1.08–4.05%) while the cluster 2 (n = 19) had samples with similar or higher levels of Fe (3.45–5.77%). Both clusters showed a very broad magnetic susceptibility ranges (cluster 1: 2.15–1003 × 10^−8^ m^3^ kg^−1^; cluster 2: 20.8–893 × 10^−8^ m^3^ kg^−1^), which suggested that there were differences in the Fe-mineralogy and geochemistry, hence in corresponding contributions to the absolute values of magnetic susceptibility in these samples. Cluster analysis could deconvolve the hidden correlations between the most important variables (of XRF signatures) and the two representative clusters will be used in further regression analyses to test the correlation with magnetic susceptibility.

### Geochemistry versus mass magnetic susceptibility of the soils from mining areas

Unlike univariate correlation which failed to yield any meaningful relationship, (e.g., Fe concentration vs soil mass magnetic susceptibility), multivariate regression (using a set of explanatory variables from the entire XRF spectra vs soil mass magnetic susceptibility) has a better chance at identifying hidden correlations between significant variables (Golia & Diakoloukas, 2022; Morrissey & Ruxton, 2018). To test for multivariate regression between XRF spectroscopic data and the mass magnetic susceptibility (*χ*) in different soils, samples originating from the three different mining areas were tested individually and as paired two-sample (coal mine) tests by PLS regression. Table 3, shows the PLS results and the main parameters obtained for both the calibration and validation datasets for each of the combinations tested. In cases where the multivariate regressions were good for both calibration and validation data sets (*R*^2^ > 0.90), XRF spectroscopic data were used alone to predict the origin of magnetic susceptibility, i.e., identifying the potential pathway for soil contamination.

**Table 3:** PLS regression parameters for the mass magnetic susceptibility (*χ*) in the soils around studied mining areas: São Pedro da Cova, Fojo, and Regoufe

Mining area	n	LV	*R*^2^		RMSE	
			cal	val	cal	val
*Individual test*						
São Pedro da Cova	49	6	0.97	0.62	44	147
Cluster 1	30	3	0.99	0.95	8.7	43
Cluster 2	19	3	0.99	0.99	8.1	14
Fojo	25	4	0.99	0.99	5.8	19
Regoufe	15	3	0.99	0.99	2.3	7.0
*Paired two-sample test*						
São Pedro da Cova—cluster 1 + Fojo	55	6	0.96	0.47	44	186
São Pedro da Cova—cluster 2 + Fojo	44	6	0.99	0.58	15	164

n, the total number of soil samples used for calibration and validation PLS regressions

**Fig. 6:**
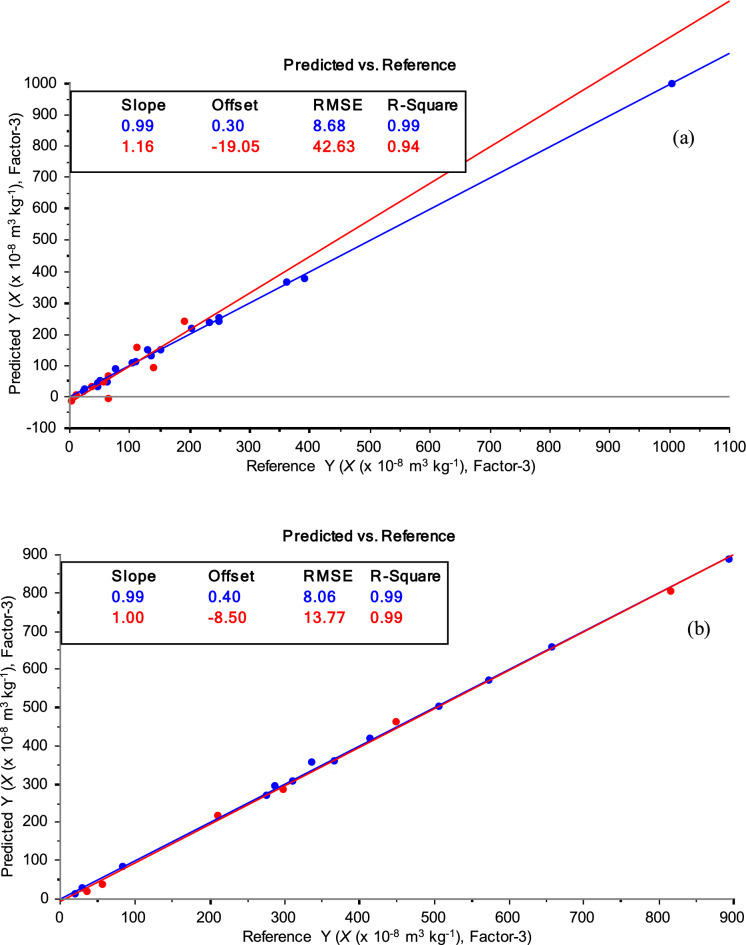
PLS regression (calibration—blue, external validation—red) between OSC-XRF spectra and mass magnetic susceptibility (*χ*) content in soil samples from São Pedro da Cova obtained after cluster analysis: cluster 1 (**a**) and cluster 2 (**b**). The model used three latent variables (factor = 3) to optimize covariance between data sets. The inserted boxes contain statistical parameters that explain the goodness of regression with high values of *R*^2^ for calibration (> 0.99) and validation (> 0.95), and low values of RMSE of calibration (equal to 8.1–8.7 × 10^−8^ m^3^ kg^−1^) and validation (equal to 14–43 × 10^−8^ m^3^ kg^−1^). Slope and offset (intercept) define the linear relationship between two variables of the regression line. The closer the slope is to 1, the data are better correlated. The offset is the intercept of the line when the X-axis is set to 0 and its value is nearly 0

Considering the individual mining areas, it can be noted that the PLS regressions gave satisfactory results for the calibration datasets in all three localities (*R*^2^ > 0.97), but the validation results were less satisfactory in samples (n = 49) from São Pedro da Cova (*R*^2^ = 0.62). This means that the calibration can be well matched within individual similar soils, but if the soil type is significantly different, the calibration may show poor results. This can be solved by making the selection of samples as similar as possible to the calibration data, or by having calibration for as many types of soil as possible. The high difference in values for calibration and validation data, can be explained mainly due to the heterogeneity of these samples, i.e., their high variability in the mass magnetic susceptibility (*χ*) caused by different factors (Ribeiro et al., 2014; Santos et al., 2023). As previously mentioned, these samples had a wide range of mass magnetic susceptibility (*χ* = 2.15–1003 × 10^−8^ m^3^ kg^−1^) not only due to particular pedogenetic factors (*i.e.*, possible higher content of ferromagnetic minerals in the host rocks) but also due to anthropogenic activities, through release of industrial waste, incineration of fossil fuel, and utilization of pesticides and fertilizers (a regular agricultural practice in urban and industrial zones of São Pedro da Cova (Santos et al., 2023). To improve the regression, a subset of samples from São Pedro da Cova: cluster 1 (n = 30) and cluster 2 (n = 19) were used (Fig. 6a, b, respectively) and regression analysis statistics gave satisfactory results (Table 3). These results confirm the previous qualitative soil clustering in which two independent groups are identified, each characterized by homogenous (similar) geochemistry. The significantly improved regressions, with higher *R*^2^ and lower RMSE for validation datasets, confirm the two subsets (cluster 1 and cluster 2) which are used for the following paired two-sample tests.

**Fig. 7:**
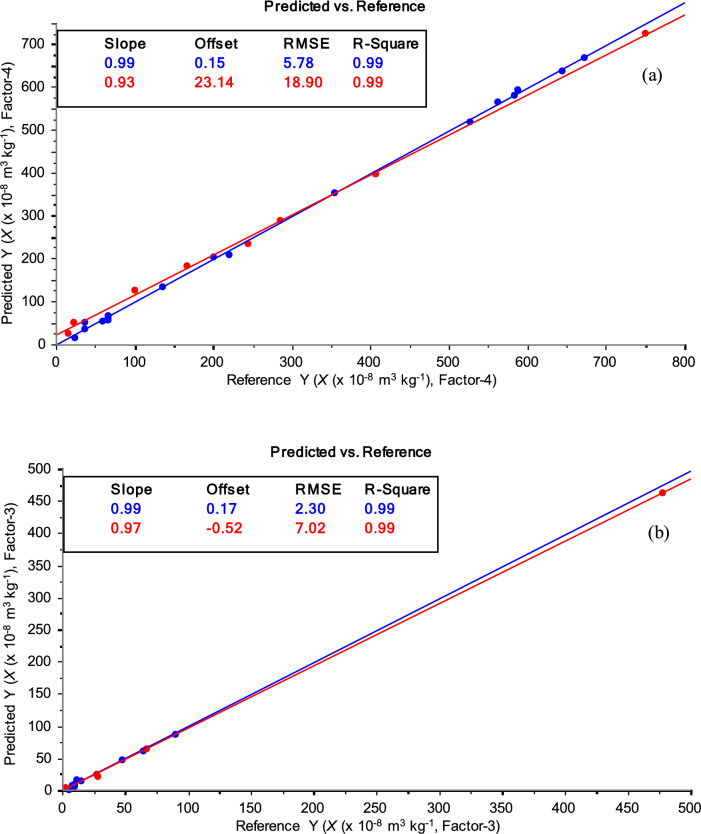
PLS regression (calibration—blue, external validation—red) between OSC-XRF spectra and mass magnetic susceptibility (*χ*) content in soil samples from: Fojo (**a**) and Regoufe (**b**). 3 latent variables (factor = 3) were used to maximize covariance between data sets. Inserted boxes contain the statistical parameters of the regression (the explanation of the relevant parameters used for statistical analysis of data is given in detail in Fig. 6)

The calibration in Fojo (Fig. 7a) or Regoufe (Fig. 7b) soil samples was confirmed by external validation results with high correlations (*R*^2^ > 0.99) and low RMSE (< 19 × 10^−8^ m^3^ kg^−1^) in these local mining areas (Table 3).

**Fig. 8:**
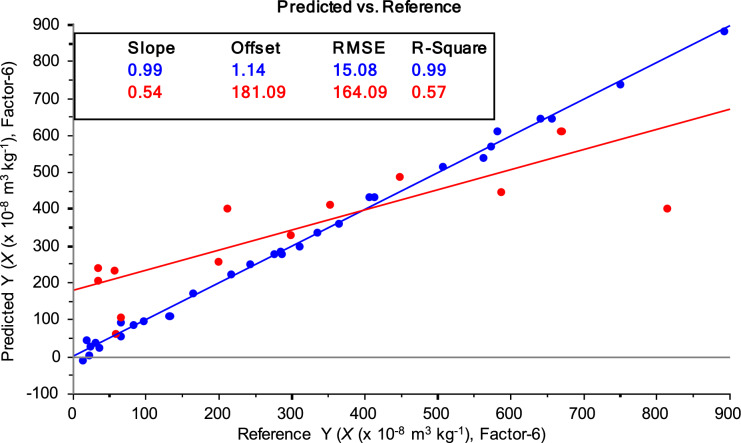
PLS regression (calibration—blue, external validation—red) between OSC-XRF and mass magnetic susceptibility (*χ*) content in soil samples from São Pedro da Cova (cluster 2) and Fojo (n = 44). 6 latent variables (factor = 6) were used to maximize covariance between data sets. Inserted box contains the statistical parameters of the regression (the explanation of the relevant parameters used for statistical analysis of data is given in detail in Fig. 6)

Regarding paired two-sample tests, after including soil samples (cluster 1 or 2) from São Pedro da Cova and Fojo sites, PLS regression could not provide a good regression to satisfy analytical purposes. Both PLS regressions were characterized by low *R*^2^ (0.47–0.58) and high RMSE (164–186 × 10^−8^ m^3^ kg^−1^) for external validation. As an example, Fig. 8 shows the regression of the combined test with São Pedro da Cova (cluster 2) and Fojo.

In the paired two-sample tests, the regressions were poor with increased errors due to the greater variety of the mass magnetic susceptibility, which implies a reduced reliability of XRF–MVA at a regional level, including samples from the two coal mining areas of different locations. The obtained PLS regression results show that the applicability of the XRF to monitor soil magnetism origin depends not only on the range of values of soil mass magnetic susceptibility (*χ*) but also on their source, *i.e.*, the environmental characteristics of each single mining area.

These results support the ability to use mass magnetic susceptibility and XRF spectral data to discrimination between soils of different origins—including influences from geological and anthropogenic processes, e.g., contamination from agricultural and industrial activities versus natural environmental inputs. The unsupervised classification MVA can successfully distinguish samples, where there are at least two specific clusters with statistically significant regressions at the local scale.

### Important variables of XRF spectra for soil magnetism assessment

**Fig. 9:**
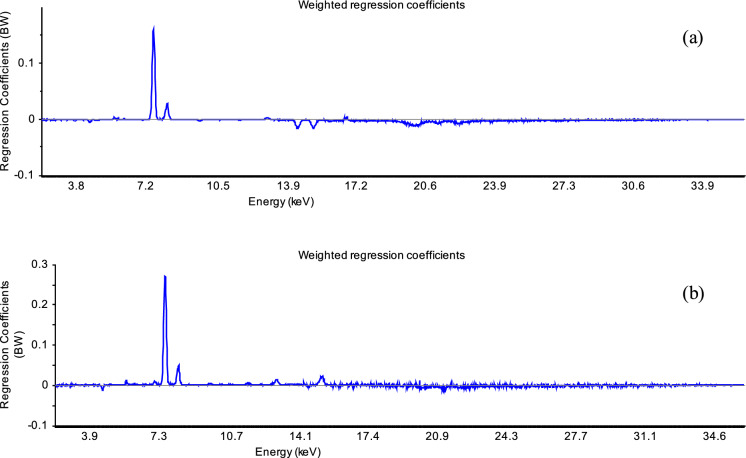
The plots of weighted regression coefficients (B_w_) obtained using 1 factor for the PLS regressions in soil samples from São Pedro da Cova: cluster 1 (**a**) and cluster 2 (**b**). The peaks with the highest regression coefficients (at 6.4 and 7.1 keV) corresponding to Fe, play the most important role for the mass magnetic susceptibility in the studied soils

**Fig. 10:**
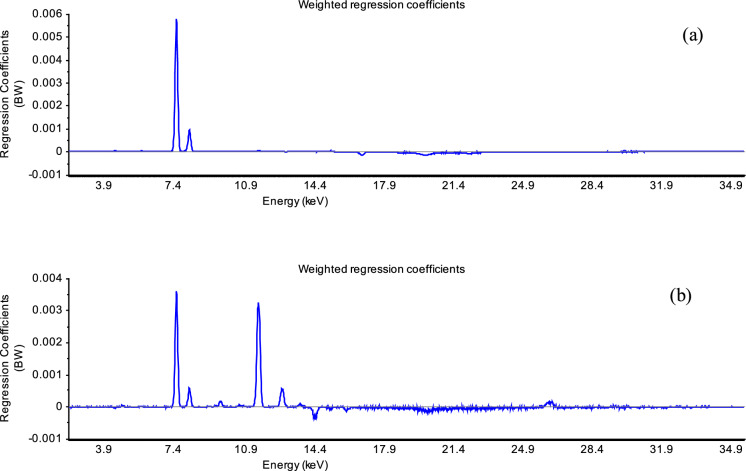
The plots of weighted regression coefficients (B_w_) obtained using 1 factor for the validated PLS regressions in soil samples from: Fojo (**a**) and Regoufe (**b**). The peaks with the highest regression coefficients (at 6.4 and 7.1 keV) corresponding to Fe, play the most important role in the regression for the mass magnetic susceptibility in the studied soils from Fojo and Regoufe. In soils from Regoufe mining area, As (10.5 and 11.7 keV) was also very important variable for the PLS regression of the mass magnetic susceptibility

The most important variables of XRF spectra (geochemical features) for the soil magnetism at local mining areas are shown in Figs. 9 and 10. As expected, Fe (6.4 and 7.1 keV) is the variable that dominantly affects the soil magnetism in all studied areas. In addition to Fe, in the regression with cluster 1 (n = 30; samples from São Pedro da Cova), Rb (13.4 keV) and Sr (14.1 keV) were negatively weighted (with small regression coefficients), showing their influence on magnetic susceptibility (Fig. 9a). For the magnetic properties of soils of cluster 2 (n = 19), Ti (4.5 keV) and Sr (14.1 keV) are important positive variables (Fig. 9b). Both clusters 1 and 2 have similar concentrations of Ti, 0.13–0.52% and 0.12–0.46%, respectively, while Sr has a higher content in cluster 1 (up to 0.07%) compared to cluster 2 (up to 0.02%). The ratios between total Fe and Ti concentrations are higher in cluster 2 (Fe/Ti = 9–48), almost twice compared to cluster 1 (Fe/Ti 3–25), which might correspond to different iron–titanium oxides, occurring as ferromagnetic minerals in the soils. Similarly, Fe/Sr ratios are as much as 2.5 times higher in cluster 2 (up to 1463) compared to cluster 1 (up to 582). These results indicate different origins for the magnetic properties due to distinct mineralogy in these subsets of soil samples, which were sampled in different zones of the mining area (Fig. 2a).

In the Fojo mining area, the mass magnetic susceptibility was slightly influenced by Zr (15.7 keV) (Fig. 10a), which is found in concentration levels from 0.006 to 0.012% in selected samples (Table 2), probably characterizing a silicate mineral phase (Ribeiro et al., 2011). These results differ from the previously identified controlling variables for the two clusters studied around the São Pedro da Cova coal mine (Fig. 9), which explains the different nature of magnetism, as well as complexity of soil magnetic properties in the individual coal mines. Also, the importance of the contribution of geochemical elements to the soil’s magnetic properties, identified by MVA, should be emphasized.

In the Regoufe W–Sn mine samples, the profile of the important variables that most affect the mass magnetic susceptibility is different (Fig. 10b). In addition to Fe, the predominant variable is As (10.5 keV and 11.6 keV) with a strong correlation with the mass magnetic susceptibility, which probably indicates the influence of arsenopyrite (FeAsS) in the mine waste soils. In these samples the concentration of As is high, ranging from 0.075% up to 1% (Table 2), which indicates mine waste contamination. Previous studies have shown that magnetic spherules of magnetite (Fe_3_O_4_), of anthropogenic origin, are the main magnetic carrier, heterogeneously distributed in the soil samples (Sant’Ovaia et al., 2023). In addition to Fe and As, the mass magnetic susceptibility is also affected by Zn (8.6 keV), Rb (13.4 keV), and Sn (25.2 keV), which are found in these samples at maximum respective levels up to 0.05%, 0.08%, and 0.01%.This confirmed the influence of pedological processes and lithological differences in the studied samples, as well as the contribution of anthropogenic magnetic spherules. These results are supported by earlier geological studies which showed that the Regoufe pluton was a muscovite-albite porphyritic granite, containing mineralized quartz veins with arsenopyrite (AsFeS) and cassiterite (SnO_2_), with minor amounts of sphalerite (ZnFeS), among other metallic minerals (Sant’Ovaia et al., 2023).

## Conclusions

The use of MVA to analyse the entire XRF spectra (including all characteristic geochemical features) to discriminate the origin of mass magnetic susceptibility in soil samples shows the relative contribution of different geochemical parameters in determining the soil magnetic properties. The XRF–MVA models were developed and validated for individual mining areas and their application to similar mining areas in other geographical zones should be tested. Importantly, the use of statistical analyses of the type used here demonstrates the linkages between magnetic susceptibility and chemical composition of the soils and the relationship to contaminant from anthropogenic processes and activities.

Wide ranges of absolute values of the mass magnetic susceptibility (2.15–1003 × 10^−8^ m^3^ kg^−1^), obtained by standard laboratory measurements in soils from three abandoned mining areas were comprehensively interpreted by the developed XRF–MVA models. The application of PCA to XRF soil spectroscopic signatures provides significant information to enable sample discrimination (PC-1 + PC-2 = 94%) and identification of soils with specific inputs from both mining activities and the natural environment, with subsequent cluster analysis discriminating between contaminated and non-contaminated soils. The PLS multivariate regression confirmed the strong positive correlation (*R*^2^ > 0.95) between soil geochemistry and mass magnetic susceptibility, selecting the most important model variables affecting soil health.

We find the applicability of the XRF–MVA models more useful at the local rather than the regional level due to differences in the sources of soil and contamination between different localities and mine types. Future research should include increased sample sizes at a more diverse range of mining areas(including more and less contaminated soils), to test the regional applicability of the XRF–MVA models with all their advantages in the assessment of specific details of soil magnetic properties. The site-specific model explained here adopts a holistic approach that can be a useful contribution to ecologists and geochemists, as well as other soil science professionals, as a case study for rapid surveys for soil contamination.

## Data Availability

All data and materials in this study are provided in the manuscript.
